# Oversight of EU medical data transfers – an administrative law perspective on cross-border biomedical research administration

**DOI:** 10.1007/s12553-017-0182-6

**Published:** 2017-03-07

**Authors:** Jane Reichel

**Affiliations:** Faculty of Law, Uppsala University, Uppsala, Sweden

**Keywords:** Privacy, Data protection, Data transfer, Medical research, Ethical review

## Abstract

The notion of privacy has long had a central role in human rights law, not least in connection to health and medicine. International, regional and national bodies have enacted a number of binding and non-binding document for physicians and researchers to adhere to, in order to protect the autonomy, dignity and privacy of patients and research subjects. With the development of new technologies, the right to privacy has gained a new perspective; the right to protection of personal data within information and communication technologies. The right to data protection has been attributed an increasing importance within EU law. Accordingly, the use of health data in medical research in general and in biobank-related medical research in particular, has made data protection law highly relevant. In medical research involving biobanks, transferring human biological samples and/or individual health data is taking place on a daily basis. These transfers involve several oversight bodies, institutional review boards (IRBs), research ethics committees, or even data protection authorities. This article investigates the role of these national oversight bodies in the transfer of health data in cross-border research, from an EU law point of view. A special focus is laid on transfer of health data for research purposes from the EU to the US, in the light of the recently enacted EU-US Privacy Shield. The main question posed is how American oversight bodies for medical research can be expected to handle the increasingly strict EU requirements for the processing of health data in medical research review.

The notion of privacy has long had a central role in human rights law, not least in connection to health and medicine [1]. International, regional and national bodies have enacted a number of binding and non-binding document for physicians and researchers to adhere to, in order to protect the autonomy, dignity and privacy of patients, research and data subjects.^1^ With the development of new technologies, the right to privacy has gained a new perspective; the right to protection of personal data within information and communication technologies. Within EU law, the right to data protection has been attributed an increasing importance. As has been pointed out by Slokenberga, EU law appears today to identify the general right of privacy and a right to data protection as two distinguishable, but connected rights.^2^ In the EU Charter of Fundamental Rights, privacy is protected under Article 7 and data protection under Article 8.

The use of health data within medical research in general and in biobank-related medical research in particular, has made data protection law highly relevant. In medical research involving biobanks, transferring human biological samples and/or individual health data is taking place on a daily basis. Several oversight bodies, institutional review boards (IRBs), research ethics committees, or even data protection authorities, may be involved in a trans-border situation.

The role of these national oversight bodies in the transfer of health data in cross-border research is the topic of this article. The question will be analysed from a EU law point of view, with the EU Data Protection Law in focus. These oversight bodies are commonly set up to review and assess the use of health data in medical research, balancing the risks and benefits raised in research projects.^3^ Opinions concerning how to balance the risks and benefits in cross-border sharing of sensitive data, such as health data, vary among different segments of society. The international biomedical research community on one hand is “advancing a paradigm that embraces the borderless use and sharing of data”,^4^ whereas the Court of Justice of the European Union (CJEU) and the EU legislator appear to be taking a more restrictive approach to data transfers. In the 2015 *Schrems-*case, the CJEU found that the Commission’s decision to enter into an agreement with the United States of America regarding conditions for data transfer, the Safe Harbor-agreement, was to be annulled since it did not guarantee the rights of EU data subjects [5]. The recently adopted decision on the EU-US Data Privacy Shield sets up a rather complex governance system to ensure EU data subjects that their EU rights will also be upheld after any transfer to the US.

The perspective of this article could arguably be seen as provincial; a localized perspective taking the view of the national oversight bodies. How can American and other national oversight bodies reviewing projects involving EU data be expected to handle EU requirements for the processing of health data in medical research? How are oversight bodies supposed to relate towards national constitutional, EU and public international law, setting the frame for what law is to be applicable in a cross-border situation? Key notions raised in this discussion are sovereignty, territoriality and jurisdiction.

The EU law on data protection is currently under transformation – the 1995 Data Protection Directive^5^ was replaced by the 2016 General Data Protection Regulation.^6^ However, the regulation will not come into force until May 2018, and during this time the directive is still in effect. The regulation is a substantial piece of legislation; there are no less than 173 recitals in the preamble followed by 98 articles. The regulation refers to the legislator either in the EU or the Member States to further enact detailed rules in specific areas. The legislative process underlying the regulation stirred up quite as sum of concerns in the biobank community, and in other areas of medical and social research, relying on health data as empirical material.^7^ The final result is still not entirely clear. With regards to the area of research, the regulation on nine occasions refers to the possibility of enacting exceptions from the general data protection requirements for research purposes,^8^ and in Article 89, specific conditions for these exceptions are laid down. Since legislation with rules governing these exceptions have not yet been enacted, as of the time of this writing, it is not possible to state in any detail what requirements EU law will place on the processing of personal data in medical research in the future. However, it is possible to analyse here, the regulation’s general direction, and to clarify the general conditions in relation to the question posed: How can national oversight bodies reviewing projects involving EU data be expected to handle EU requirements for the processing of health data in medical research?

The issues relating to international transfers of medical data are relevant to most medical research, even though the greater numbers of examples here are found in the area of biobanking. The reason for this is more a matter of practicality; this author has been involved in several EU-projects relating to biobanks in recent years.^9^

This article is structured as follows. Section 1 provides a brief overview of the relevant bioethical principles and laws. The concepts of sovereignty, territoriality and jurisdiction are introduced in section 2. The responses in EU law towards the legal challenges posed to cross-border administration are discussed in section 3. The article concludes with an analysis of consequences for national oversight bodies, and to whether national oversight bodies are to become gatekeepers of foreign rights or rather, as the biomedical community would prefer, global standards, section 4.

## Privacy and autonomy in biomedical research – rules and governance structures at a glance

There are two basic points of departure in legal and bioethical doctrine in regards to the treatment of human biological samples required for research, which has reached almost universal acceptance. First, the use of human biological samples in research is conditional on eligible donors providing informed consent to such use. Second, that research on human biological samples are to be placed under the review of independent research ethics committees.^10^ There are scarcely any binding international conventions at the global level that directly address these issues,^11^ but they can be considered indirectly covered by the UN Declaration on Human Rights^12^ and further targeted in several international soft law documents.^13^ In Europe, both the Council of Europe^14^ and the EU have enacted binding rules. The EU has included explicit rules for both handling human biological samples, and the handling of personal data, in the EU Charter, requiring an informed consent from the sample donor/data subject, or, in regards to data, some other legitimate basis laid down by law.^15^

To ensure the correct implementation of the principles and rules associated with medical research, national oversight bodies, ethical research committees, are called upon to give an ethical approval for any research project comprising the handling of human biological samples.^16^ All of the above-mentioned guidelines and recommendations require the involvement of research ethics committees in some form.^17^ Ethical approval is generally needed when setting up a biobank, collecting samples (and personal data) for a specific research project, or when re-using old samples already stored in biobanks [11].

There does not seem to be an equivalent consensus regarding the role of research ethic committees in connection to data protection in research. Unlike human biological samples, the transfer of health data is highly regulated even outside medical research. In EU law, health data belongs to a special category of data, referred to as sensitive data, where the requirements for processing are set even higher.^18^ EU law thus requires that Member States allow for the processing of health data in research only when ‘suitable safeguards’ or, agreement with the specified terminology in the General Data Protection Regulation such as ‘appropriate safeguards’ are in place.^19^ An appropriate safeguard could however be the involvement of a research ethics committee, such as for example in Swedish law.^20^ In practice, research ethics committees thus play a very central role in the process of launching medical research projects, without which the research cannot be conducted.^21^

EU data protection law further requires explicit informed consent for handling of the health data, even if exceptions for processing for research are available.^22^ EU data protection law also gives the data subjects several other rights in connection to the processing of their personal data, for example a right to information of how the personal data is being processed, a right to rectification of the data and right to a judicial remedy for any breaches of these rights.^23^ Both the current directive applied today and the Regulation in effect as of 2018 allow for certain exceptions from informational rights and the right to rectification in relation to the processing of health data in research.^24^ As discussed further below in section 3, the application of data protection law on medical research is not always clear and straightforward. This may be especially true for oversight bodies outside the EU, who, according to the long reach of EU data protection law could be tasked with upholding EU data protection rights in their native countries.

## Sovereignty, territoriality and jurisdiction

A basic feature of the classical (yet contested) understanding of the concept of state sovereignty, according to Slaughter, is the right to be left alone, to have a sphere where other states cannot interfere with the internal affairs of the state.^25^ She refers to this notion being included in Article 2 (7) of the UN Charter; “Nothing contained in the present Charter shall authorize the United Nations to intervene in matters which are essentially within the domestic jurisdiction of any state.” Classical public international law thereby builds on the premise that no state can be bound to follow rules to which it has not consented, i.e., consensual rulemaking.^26^ Connected to state sovereignty are the principles of self-determination and territoriality. These principles entail that it is the task of each state and their respective administration to handle administrative issues concerning the state, its citizens and activities as carried out within the state borders.^27^ Within its territory, each state has jurisdiction over any legal matters occurring there. The public international law of jurisdiction is thereby used to ensure that states do not assert jurisdiction over affairs that are the domain of another state.^28^ A state will thus not be bound by legal acts enacted outside that state, neither administrative decision nor court judgments, unless it has so agreed through international agreements, treaties or conventions.^29^

The understanding of sovereignty, territoriality and jurisdiction may differ between different fields of law. The laws on jurisdiction stem from criminal law and even though its principles can be applied generally, certain adaptions may be necessary.^30^ In the field of administrative law, there traditionally has been a strong connection between the law and the development of the modern nation state.^31^ The main task of the administration, as part of the executive branch, is to implement policies enacted by its own national legislator.^32^ It therefore has not been a very common situation for a national authority to implement policies from a foreign state, for example, by scrutinizing whether bioethical principles and laws are upheld within a research project.

Through the current processes of globalization and technical development, not least with respect to the Internet, the traditional understanding of the concepts of sovereignty, territoriality and jurisdiction have been placed under pressure. Slaughter has identified two fundamental challenges to the concept of sovereignty.^33^ First, the ineffectiveness challenge, referring to that “a state’s ability to control its own territory without external interference is no longer sufficient to allow it to govern its people effectively – to provide security, economic stability, and a measure of prosperity, clean air and water and a minimum of health standards.”^34^ Second is the interference challenge, “[a]ll human rights”, Slaughter maintains, “deliberately infringe on domestic jurisdiction of every state, denying governments the freedom to torture, murder, ‘disappear’ or systematically discriminate against their own citizens”.^35^

The understanding of administrative law as a traditionally domestic area of law has also undergone changes. Schmidt-Aßmann has stated that the “internationalization of administrative activity can be defined as processes of an administrative nature extending beyond national administrative borders, either because they have evolved beyond such borders or because they were, from the outset, conceived without consideration of such borders.”^36^ In many areas of administrative law, such as control over pharmaceuticals and medical research, administrative cooperation beyond the state today takes place - on a daily basis.^37^ The legal ground for this cooperation is normally laid down in international conventions, treaties and agreements, where the participating states have agreed to waive some of their sovereign rights in order to be part of a mutually beneficial cooperation. The EU is an example of this, a treaty-based international organization, today with considerable “constitutional” traits. The administrative cooperation framework between EU and national authorities has been developed so far that it may - in certain areas of law and to a certain extent– be considered a supranational administrative organization in itself [25].

In connection to those administrative law areas that are also covered by human rights law, further considerations need to be taken into account. As pointed out by Slaughter, human rights requirements per se constitute an intrusion into the sovereignty of the state, in that the state cannot regulate, nor act in violation to, internationally binding law. Further, regional fundamental law requirements may hinder a state from entering into agreements with other states, not adhering to the same level of protections within a specific field of law. This is evidently the case with data protection in Europe, where both the Council of Europe and the EU set out requirements at a level above and beyond the level of protection in other states.^38^

## Administrating EU data transfers in cross-border research

As stated above, the EU arguably has the strictest data privacy law in the world, entailing a certain strain on the transfer of data outside the Union, especially since the EU has the ambition of upholding data privacy rights for EU subjects even when the data is processed overseas. In medical research based on biobanks, both human biological samples and personal data on health are used.

Some introductory remarks are given in this section on the relationship between samples and data in order to specify what resources are covered by the EU data protection law (section 3.1). The long reach of EU data privacy law is then discussed in section 3.2. These rules are analysed in section 3.3 in a medical research setting.

### Data and samples

The starting point when applying data protection law is defining data. Can a human biological sample itself be considered data? Both the Data Protection Directive and the General Data Protection Regulation define personal data as “any information relating to an identified or identifiable natural person”.^39^ Neither act defines more closely what type and form ‘the information relating to an identified, or identifiable natural person’ is to take. According to Article 29 of the Data Protection Working Party Group, human biological samples, such as blood samples, are themselves sources from which data is extracted, but they are not data in themselves; “the extraction of information from the samples is collection of personal data, to which the rules of the Directive apply. The collection, storage and use of tissue samples themselves may be subject to separate sets of rules.”^40^

The preamble to the General Data Protection Regulation states that “[g]enetic data should be defined as personal data relating to the inherited or acquired genetic characteristics of a natural person which result from the analysis of a biological sample from the natural person in question, in particular chromosomal, deoxyribonucleic acid (DNA) or ribonucleic acid (RNA) analysis, or from the analysis of another element enabling equivalent information to be obtained.^41^ Once again, the analysis of the biological sample is considered to be data, but not the sample itself. The sample is thus not protected under EU Data Protection Law.

Consequently, EU data protection law is highly relevant to medical research: the result from a DNA analysis will always be considered personal data, even without any accompanying information such as the name of the patient or a code to which only trusted third parties have the key. The DNA in itself is an identifier. However – even if EU data protection law is highly relevant, it is not alone. As pointed out by the Article 20 of the Data Protection Working Party Group, the handling of human biological samples may be subject to other sets of rules.

### The long reach of EU data protection law – extraterritorial scope and strict requirements for transferring data

All states by definition are, at least as a point of departure, sovereign and thereby independent of each other. One feature of sovereignty is the capacity to decide on legal matters occurring within the borders of that state. Legal conventions on jurisdiction have been seriously challenged by the types and forms of data usage within the Internet.^42^

Within its data protection law, the EU has taken as a general standpoint that the rights of EU data subjects should be protected regardless of where the data is processed. The EU has thus established two different paths to ensuring that EU data subjects’ rights to data protection are not undermined by free-flowing information routes on the Internet. First, the scope of application of the EU data protection law is very wide and could even be described as extraterritorial (section 3.2.1). Secondly, EU data protection law sets up far reaching requirements on the recipient of EU data in order for a transfer of data outside the scope of application of EU data protection law to be considered legal (section 3.2.2). These rules have especially stirred some controversy in relation to the US, as analysed in section 3.2.3.

#### The territorial scope of application of EU data protection rules

The Data Protection Directive already had an unusually broad definition of territorial scope,^43^ and the General Data Protection Regulation expands it a bit further. Article 3.1–2 of the Regulation states:

1. This Regulation applies to the processing of personal data in the context of those activities of an establishment of a controller or a processor in the Union, regardless of whether the processing takes place in the Union or not.
2. This Regulation applies to the processing of personal data of data subjects who are in the Union by a controller or processor not established in the Union, where the processing activities are related to:
  - the offering of goods or services, irrespective of whether a payment of the data subject is required, to such data subjects in the Union; or
  - the monitoring of their behaviour as far as their behaviour takes place within the Union.

According to the first paragraph, all situations where a person who is established in the EU processes personal data, either on one’s own behalf (controller) or on behalf of another (processor),^44^ must follow the rules in the regulation. Whether the data is actually processed, for example, in a cloud service, is irrelevant. This means, for example, that if a medical researcher responsible for processing personal data in Japan (a controller, in EU terms) uses a data storage service established in the EU, (a processor established in the EU), the Japanese controller must adhere to EU law in regards to the data stored with the European service provider.

The second paragraph defines certain situations in which it is the EU data subject him or herself that renders the regulation applicable, even when the controller and processor is established outside the EU. This is the case where the controller or processor targets the EU data subject, either by offering goods or services, or by monitoring their behaviour, if the behaviour takes place within the EU. There are countless providers of services and goods all over the world who may fall under this category, requiring them to uphold EU data protection law.^45^

#### The requirements for transferring data to third countries

Both the Data Protection Directive and the General Data Protection Regulation contain rules setting out the requirements and conditions for the transfer of personal data outside the EU. Content-wise, the differences between the directive and regulation are not significant, however, the “rules” within the regulation are more detailed.

Article 44 of the General Data Protection Regulation sets out the general principles for allowing transfers to third countries, including any onward transfer:

Any transfer of personal data which are undergoing processing or are intended for processing after transfer to a third country or to an international organisation shall take place only if, subject to the other provisions of this Regulation, the conditions laid down in this Chapter are complied with by the controller and processor, including for onward transfers of personal data from the third country or an international organisation to another third country or to another international organisation.

All transfers outside the EU must first comply – as always - with the principles in the regulation, meaning that there has to be a legal basis for the processing of the personal data that includes a transfer outside the EU.^46^ In regards to special categories of data, such as health data, there must further be a specific legal ground for processing.^47^ Secondly, there has to be mechanisms in place to ensure that the rights of the EU data subjects will also be upheld when the data is transferred outside the EU.

The regulation provides a number of set procedures that can be divided into three main categories. These can be seen as hierarchical, with the first category offering the most efficient and thorough protection. First, transfer may take place if the Commission has enacted an *adequacy decision*, meaning that the Commission has found that “a country, a territory or one or more specified sectors within that country… ensures an adequate level of protection” (Article 45).^48^ Protection in this context refers to legal protection, the existence of a legal framework for data protection, together with sufficient enforcement mechanisms. A Safe Harbor Agreement, further discussed in section 3.2.3, is an example of this [27]. Secondly, in the absence of such decision, data may be transferred if *appropriate safeguards* are available, on the condition that enforceable data subject rights and effective legal remedies for data subjects are available (Article 46).^49^ These safeguards, for example, may be legally binding and enforceable instruments between public authorities, binding corporate rules (later further regulated in Article 47), standard data protection clauses adopted by the Commission, or on the basis of especially approved codes of conduct. Contractual clauses between the sender and recipient, subject to the authorization of a competent supervisory authority, also fall within this category. Thirdly, in the absence of either an adequacy decision or appropriate safeguards, there is a list of *derogations* in Article 49.^50^

There are two categories of derogations; Article 49.1(1)(a)-(g) lists seven specific situational derogations. One is the existence of an explicit informed consent from the data subject where he or she has been informed of the possible risk of transfer. Others are where the transfer is necessary due to a contract involving the data subject, an important reason of public interest or in connection to a legal claim. Article 49.1(2) contains the second category of derogations, which is open, but can only be used under rather limited circumstances:

[O]nly if the transfer is not repetitive, concerns only a limited number of data subjects, is necessary for the purposes of compelling legitimate interests pursued by the controller which are not overridden by the interests or rights and freedoms of the data subject, and the controller has assessed all the circumstances surrounding the data transfer and has on the basis of that assessment provided suitable safeguards with regard to the protection of personal data.

The controller must further then inform the competent supervisory authority, as well as the data subjects concerned.

A fourth and final procedure is mentioned only briefly here; the transfer of judgments and official decisions, requiring a controller or processor to disclose personal data, is permitted if based on an international agreement, such as a mutual legal assistance treaty (Article 48). If none of the abovementioned grounds are available, the transfer of EU data outside the Union is not allowed.

#### The Safe Harbor-agreement, Schrems and the EU/US privacy shield

The Safe Harbor-agreement between the EU and US is an example of an *adequacy decision*, enacted under Article 25.6 of the Data Protection Directive, the predecessor of Article 45 in the General Data Protection Regulation.^51^ The agreement itself was annexed to an actual decision enacted by the Commission, ensuring that entities within the US adhered to the principles laid down in the agreement. Thereby the entities could be considered trustworthy recipients of EU data. EU controllers could transfer personal data to these entities without further ado. As stated above, the Court of Justice in *Schrems* found that the Commission’s decision was invalid, since the Safe Harbor agreement did not sufficiently ensure an adequate level of protection for EU data rights.^52^

The *Schrems-*case was brought by an Austrian law student, Maximilian Schrems. He argued that his personal data on his Facebook account was not properly protected, a fact made apparent following the revelations made by Edward Snowden concerning the activities of the US intelligence services.^53^ The Court held that while the term “adequate level of protection” in Article 25(6) of Directive 95/46/EC does not mean a level of protection identical to that guaranteed in the EU legal order, it must be understood as requiring a third country to ensure a level of protection of fundamental rights and freedoms “essentially equivalent” to that guaranteed within the Union by virtue of the Data Protection Directive, read in light of the EU Charter of Fundamental Rights.^54^

The main criticism of the Court focused on the obligation for entities within the US to disregard the Safe Harbor-principles, in the event “national security, public interest, or law enforcement requirements” within the US legal system so required.^55^ As the Court rather laconically stated, since US legislation permitted public authorities such as the NSA “to have access on a generalised basis to the content of electronic communications”, this constituted a breach of the EU right to privacy as guaranteed by Article 7 of the Charter.^56^ There is no legal basis in EU data protection law that would render such indiscriminate surveillance of personal data lawful. Further, the fact that the US legal system did not provide for any effective remedy for EU data subjects was contrary to Article 47 of the EU Charter and the right to effective judicial protection.^57^

The legal protection sought after by the CJEU did thus not find its match within the political reality that the Commission had tried to master over the years of negotiations with its American counterpart. The *Schrems –*case was not the first time criticism towards the US was heard from the EU. Negotiation between the Commission and the US on different aspects of data protection had been going on already since 2010,^58^ and were intensified after the verdict of the Court was delivered in 2015. A political agreement between the EU and US was reached in early in 2016,^59^ and the Commission presented a communication to the EU legislators at the end of February, explaining the agreement in detail.^60^ A new adequacy decision, the EU-US Privacy Shield, was enacted in July 2016.^61^

The EU-US Privacy Shield is drafted as a general decision on the transfer of data, but is mainly directed to commercial activities and businesses. The Privacy Principles comprise thirteen Framework Principles similar to those in the Safe Harbor-agreement. Further there are Supplemental Principles, including specifications and exceptions to the framework principles as well as informational and institutional rules for the American data controllers to follow. The principles are found in Annex II to the draft decision.^62^

That more interesting for the issue raised here is the governance structure of the agreement and the requirements to guarantee recourse mechanisms for EU data subjects. The EU-US Privacy shield is built on a system of self-certification by which US organizations commit to the Privacy Principle.^63^ The US Department of Commerce maintains, it is to maintain a list of all participating US organizations that have committed to the principles. In order to remain on the list, organizations will have to re-certify annually.^64^

Further, under the Recourse, Enforcement and Liability Principle, all participating listed organizations must provide “robust mechanisms to ensure compliance with the other Principles and recourse for EU data subjects whose personal data have been processed in a noncompliant manner, including effective remedies”.^65^ Organizations may choose independent recourse mechanisms in either the EU or US. This includes the possibility to voluntarily commit to cooperating with the European data protection authorities. If handling human resources data collected in the context of an employment relationship, cooperation with EU authorities is mandatory.^66^ This also affects the choice of applicable law, since EU law will be relevant for interpretation of the compliance of US organization^67^:

U.S. law will apply to questions of interpretation and compliance with the Principles and relevant privacy policies by Privacy Shield organizations, except where such organizations have committed to cooperate with European data protection authorities.

European data protection authorities are to establish a specific pan-EU panel to resolve these complaints.^68^ When US organizations are cooperating with EU data protection authorities, the US actors will accordingly have to abide by the EU interpretation of the Privacy Shield and its principles and will further be bound by the decisions of a pan-EU panel.^69^

### Applying EU data protection rules on transfer of health data in medical research

How can these requirements concerning the transfer of EU data be applied in the context of biomedical research? As set out above, a transfer of a human biological sample includes privacy issues both regarding the sample itself and regarding the personal data retrievable from the sample. These two issues are dealt with separately, at least within the EU. Regarding the transfer of the biological material itself, there are no globally applicable administrative rules (section 1). According to established medical research practices, a transfer of human biological material is to be preceded by entering into an agreement between the sender and recipient, a Material Transfer Agreement (MTA).^70^ All the conditions for handling the samples are regulated in the MTA, such as specific restrictions regarding the given consent, etc.^71^

Transfer of data within medical research from the EU must fall within one of the mechanisms set out above (section 3.2.2.) Even before the *Schrems*-judgment, the Safe Harbor principles were not commonly used when transferring health data to medical researchers in the US, since the principles are directed towards commercial activities and not research. Instead, an appropriate safeguard mechanism was applied, contractual clauses having been authorized by a competent supervisory authority, i.e. a research ethics committee.^72^ These contracts are referred to as Data Transfer Agreements (DTA), regulating the obligations of both the sender and recipient of the data.^73^ It should however be underlined that the requirements laid down in Safe Harbor, the *Schrems-*case and now EU-US Privacy Shield are still relevant for medical research, since they can be used as benchmark as to what level of protection should be ensured EU data subjects.

As stated above, applying an appropriate safeguard in the General Data Regulation is conditioned on the availability of “enforceable data subject rights and effective legal remedies for data subjects”.^74^ This condition is not explicitly laid down in the Data Protection Directive. Neither does the directive explicitly require this in reference to the assessment for an adequacy decision.^75^ The Court placed considerable weight on these issues in *Schrems* 76:

Even though the means to which that third country has recourse, in this connection, for the purpose of ensuring such a level of protection may differ from those employed within the European Union in order to ensure that the requirements stemming from Directive 95/46 (Data Protection Directive] read in the light of the Charter are complied with, those means must nevertheless prove, in practice, effective in order to ensure protection essentially equivalent to that guaranteed within the European Union.

Since the US legal system could not ensure this protection, it was not considered safe. As pointed out by Hofmann, this is the first time the Court has declared an EU act illegal due to breaches of fundamental rights without performing a balancing test to assess whether the limitation of the fundamental right could be seen as legitimate in a democratic society.^77^ The breach of the right of private life and to an effective judicial review was consequently so far-reaching that it was seen as violating the “essence both of the right to privacy and the protection of personal data as it arises from Articles 7 and 8 of the Charter as well as the essence of the right to an effective judicial remedy under Article 47 Charter”.^78^

It remains to be seen how the more detailed rules for processing of health data in research will be regulated in the EU Member States. Arguably, the focus on the rights of the data subject may entail a shift concerning the expected mandate of the research ethics committees and other oversight bodies for research, if they are to uphold the Data Protection Regulation. As of today, the specific rights of the data subject, amongst others involving such matters as the right to redress, is not usually addressed in either MTAs nor DTAs entered into by sending and receiving research institutions. These agreements often include how to handle legal conflicts between the sending and receiving research institutions, for example issues relating to intellectual property, but the sample donor/data subject is usually absent.^79^ The research ethics committees within the EU which are approving DTAs for transfers to third countries should thus ensure that there are effective remedies available for the data subject within the receiving institution. Accordingly, research ethics committees in states outside the EU could be asked to review and ensure the rights of EU data subjects in order to allow for research cooperation. The requirements within EU law create new tasks to be handled within the governance structure for applicable bioethical aspects. Whether this is good is discussed in the following and final section.

## Research ethics committees as gatekeepers of foreign rights or global standards – what about the localized administrative law perspective?

Returning now to the localized administrative law perspective perspective of research oversight; what should be expected of national research ethics committees? As seen above, the core task for these committees is to assess research proposals from the point of view of a risk-benefit analysis; do the benefits of the proposed research outweigh the potential risks for research subjects or others? This task is not conducted in a vacuum; the research committee is embedded in constitutional, societal and cultural contexts. These contexts in turn are connected to notions such as sovereignty, territoriality and jurisdiction, as such can be understood in a globalized world. The notions are not the same as they used to be, but they are not yet obsolete. States and regional international organizations such as the EU still have an interest in ensuring that the legal rights enacted to protect their citizens (and others) can be effectively implemented. The issue at stake here is that with globalization and the internet, states and international actors are no longer able to single-handedly command and control the arena in which rights are to be upheld. This is especially true with the rights to data protection.

Even within the globalized area of cross-border medical research, states and regional international organizations are thus relevant. The underlying values and ideas of the bioethical aspects of law can to a large extent be described as universal, but there are still national and regional differences, not the least when it comes to data protection. In the Helsinki Declaration, the connection between the research ethics committee and the law of the land is underscored but not given exclusive importance^80^:

It [the research ethics committee] must take into consideration the laws and regulations of the country or countries in which the research is to be performed as well as applicable international norms and standards but these must not be allowed to reduce or eliminate any of the protections for research subjects set forth in this Declaration.

The law of the land is to be applied, together with relevant international norms and standards, as long as these do not undermine the Helsinki Declaration itself. The question here is what happens if the connections to the constitutional, societal and cultural contexts are altogether detached, as in the situation where a research ethics committee is to uphold the rights enacted by a legislator in another part of the world. According to EU data protection law, a transfer of data is conditioned on the fact that the receiving institutions can guarantee the data subject that his/her rights according to EU law will be protected to an acceptable degree.^81^ EU data rights are to be respected regardless of where the data is processed in the world.

There is an obvious risk of an “over-bureaucratization” of data protection when oversight bodies are to uphold the rules of a legislator different than the one appointed by the constitutional context in which they are embedded. Ensuring that authorities implement the rules of their own legislator in a correct and efficient manner can be challenging in itself, and to achieve the efficient implementation of rules enacted by a different legislator may need extra attention [29]. In order to verify that the data will actually be protected after a transfer, the EU /US privacy law, for example, has introduced a rather complex scheme of annual self-evaluations for companies, requirements of information, together with mechanisms of redress for data subjects. The system further is to be under the supervision of either EU and US authorities, where the allocation of the final say in interpretation of the rules may vary.^82^ However, even if the system is complex, it does not necessarily mean it is effective. It remains to be seen if the CJEU will find that the EU-US Privacy Shield affords an adequate level of protection, if and when the question will be placed before the Court.

The foreign oversight bodies tasked with supervising the processing of EU data within their jurisdiction, research ethics committees as the case may be, will be in a situation where data from individuals from the EU is to be treated differently from other individual’s data. The idea has a certain distasteful European ethno-centric ring to it. Also within the EU, the rules on transfer may to risk lead to different treatments based on the origin of the data. When importing foreign data into the EU, the equivalent protection is to be upheld vis-à-vis data subjects in third countries. However, this question has not rendered much interest from EU policy-makers or academics. What are the mechanisms in place within the EU to ensure that data subjects in third countries have consented to the use of their data, on equal terms as data subjects within the EU? How can their rights to information and access to effective judicial review be upheld? EU law does not provide any convincing answer to these queries.

The question boils down to if there is a better way. The introduction above referred to a debate within the bioethical research community, “advancing a paradigm that embraces the borderless use and sharing of data”.^83^ Those authors drew on the work done within the Global Alliance for Genomics and Health.^84^ The Global Alliance, founded in 2013, connects organizations in health care, research and secure disease advocacy, having as its mission to “accelerate progress in human health by helping to establish a common framework of harmonized approaches to enable effective and responsible sharing of genomic and clinical data, and by catalyzing data sharing projects that drive and demonstrate the value of data sharing.”^85^ In a similar vein, Dove has also argued the following in relation to biobanks^86^:

Most critically, data sharing must occur in an environment whereby the privacy interests of research participants are safeguarded throughout the lifecycle of a biobank initiative, and regardless of the location where the data are processed. Such safeguards can best be instituted where there is a global governance framework that provides substantially universally acceptable assurance that reasonable expectations of privacy will be met, and mutual recognition of the privacy norms in relation to the contemplated uses of data (and biospecimens).

The follow-up question is how to define “reasonable expectations of privacy” at the global level. The doctrine itself emanates from US constitutional law, where it is used as a test to define the scope of application of privacy protection of the Fourth Amendment to the US Federal Constitution [32]. It is quite clear at least that the EU and US have different views on data protection, judging from the *Schrems*-case, the EU-US Privacy Shield and the detailed rules on data transfer in the newly enacted General Data Protection Regulation. It is far from self-evident that data subject in the US and the rest of the world have the same expectations. Instead, a lack of trust and understanding of the others’ views on data protection seem to be a significant part of the problem.^87^

So, even if the idea of a global standard in itself sounds persuasive, it is difficult to see how it could be implemented in practice. The research ethics committees remain in a difficult situation. In this connection Kaye has maintained^88^:

The authority of these bodies are national, yet in the context of the increasingly global research, such bodies adjudicate on complex issues associated with international data sharing and privacy. National bodies do not always have the authority, scope or expertise to assess the privacy risks associated with global data sharing or to ensure compliance with their decisions.

The task is not easy, even without any consideration of the EU data protection requirements placed on non-EU oversight bodies. Yet, research ethics committees have become essential building blocks in order to achieve legitimacy and trustworthiness in medical research.^89^ Their role and function ought to be facilitated, not overburdened.

The most workable way forward may be, as often is the case, the middle way. Standard setting and self-regulation for a long time has been an important component in the governance of biomedical research [36]. A soft bottom-up approach to standard-setting and best practices within the research community in combination with national oversight bodies collaborating in mutual respect for each-others mandate and jurisdiction is more plausible. Over a longer perspective, the EU should work together with its partners in global research to facilitate the work of research ethics committees, within the EU and elsewhere, wherever EU data is processed.^90^ Until a common understanding can be reached, and such may take a considerable amount of time, the research community would do best by providing their expertise through persuasive authority in dialogues with policy-makers around the globe.

## Footnotes

1 See section 1.

2 Slokenberga, [2] Chapter 4.

3 For the sake of clarity, oversight bodies tasked with reviewing research ethics in medical research are here also referred to as research ethics committees. See further Whitley, [3] p. 39.

4 Dove, Knoppers, and Zawati, [4] p.6.

5 Directive 95/46/EC of the European Parliament and of the Council of 24 October 1995 on the protection of individuals with regard to the processing of personal data and on the free movement of such data.

6 Regulation (EU) 2016/679 of the European Parliament and of the Council on the protection of natural persons with regard to the processing of personal data and on the free movement of such data, and repealing Directive 95/46/EC (General Data Protection Regulation).

7 See for example, Abbott, [6].

8 See Articles 5.1 b and e, 9, 14, 15, 16, 17, 18 and 21 of the General Data Protection Regulation.

9 BBMRI.se, see further BioBankCloud, biobankcloud.com, and most recently, B3Afica, b3africa.org.

10 Ruffert and Steinecke, [7] pp. 94–96. The content of this section has previously been discussed in Reichel, [8], p. 172.

11 The UN has enacted the International Covenant on Civil and Political Rights (ICCPR) 1966, which targets medical or scientific experimentation, but most likely not research on samples of biological material in a biobank, see the General Comment No. 20 Replaces General Comment 7 concerning prohibition of torture and cruel treatment or punishment (Art. 7) (Mar. 10, 1992).

12 Article 1 on human dignity could be considered relevant.

13 See for example:

- Council for Int’l Organizations of Med. Sciences & WHO, International Ethical Guidelines for Epidemiological Studies, guideline 2 (2002);
- Council for Int’l Organizations of Med. Sciences & WHO, International Ethical Guidelines for Biomedical Research Involving Human Subjects (2002);
- Declaration of Helsinki Ethical Principles for Medical Research Involving Human Subjects (2008),
- UN Guidelines concerning computerized data files (1990);
- Council of Europe Recommendation Research on Biological Material of Human Origin (2006); and
- OECD Guidelines governing the Protection of Privacy and Transborder Flows of Personal Data (2013), [C (80)58/FINAL, as amended on 11 July 2013 by C (2013)79].

There is also ongoing work within several research communities to enact standards, for example, the Global Alliance for Genomics and Health, available at genomicsandhealth.org/node/12703 and the work within the BBMRI-ERIC, especially its ELSI common service. See further Rynning, [9] and Reichel, [8].

14 The Council of Europe has enacted several documents that can be cited in general terms, the European Convention for the Protection of Human Rights and Fundamental Freedoms (1950) and the Social Charter (1961, revised and expanded in 1996). More specifically, the Convention for the Protection of Individuals with regard to Automatic Processing of Personal Data Council of Europe (1980) and the Council of Europe Convention on Human Rights and Biomedicine and its Additional protocol concerning Biomedical Research. There are also soft law tools, for example Council of Europe Recommendation No. Rec (2006) 4 of the Committee of Ministers to Member States on research on biological materials of human origin.

15 Articles 3.2 and 8.2 of the EU Charter.

16 Ruffert & Steinecke, [7] p. 98.

17 See for example Guidelines 2 and 3 in Council for Int’l Organizations of Med. Sciences & World Health Org. [WHO], International Ethical Guidelines for Epidemiological Studies, (2002); Guidelines 2 and 3 in Council for Int’l Organizations of Med. Sciences & WHO, International Ethical Guidelines for Biomedical Research Involving Human Subjects, 2002, Article 9 in the Additional protocol to the Convention on Human Rights and Biomedicine, Concerning Biomedical Research, (2005), and Article 23 in the WMA Declaration of Helsinki Ethical Principles for Medical Research Involving Human Subjects, 2008. For an overview of national legal orders in Europe, see Eleni Zika et al., [10].

18 All processing of the special categories of data is prohibited unless a specific legal ground is available; Article 9 General Data Protection Regulation and Article 8 Data Protection Directive.

19 Articles 8 Data Protection Directive and Article 89 in the General Data Protection Regulation.

20 See for example in Swedish law, section 19 *Personuppgiftslag* (1998:204) [Personal Data Act, implementing the EU data Protection Directive], which refers to lag (2003:460) *om etikprövning av. forskning som avser människor* [Act concerning the Ethical Review of Research Involving Humans]. Since the requirements in the General Data Protection Regulation 2016/679 will not be implemented until May 2018, it is still unclear what the condition the ‘appropriate safeguards’ will entail at the national level. See further section 3.2.

21 See further [4, 12, 13] and section 5.

22 See Article 8 Data Protection Directive and Article 9 and 89 General Data Protection Regulation. For a comparison on the right to have an informed consent for the handling of human biological samples, and the rights included in European data protection law (including also the Council of Europe), see also Slokenberga, [2] chapter 8.

23 Articles 10–11, 12 and 22 Data Protection Directive and Articles 11–13, 16–17 (also including a right to be forgotten) and 79 General Data Protection Regulation.

24 The general requirements for exceptions are laid down in Article 8 Data Protection Directive and Article 89 in the General Data Protection Regulation.

25 Slaughter, [14] p. 284

26 Crawford, [15] p. 386.

27 Wenander, [16] p. 49, with further reference to Schwarze, [17].

28 Ryngaert, [18] p. 6.

29 Wenander*,* [19], p. 762.

30 Compare Ryngaert, [18] p 2. See also Svantesson [20].

31 Loughlin, [21] p. 437.

32 Cassese, [22] p. 605.

33 Slaughter, Sovereignty and Power in a Networked World Order, [14] p. 284.

34 Ibid.

35 Ibid.

36 Schmidt-Aßmann, [23] p. 2063.

37 See amongst other volumes [24].

38 Svantesson holds that the EU has the most influential and arguably strictest data privacy laws in the world. Svantesson, [26] p. 55. See also Slokenberga, [2] chapter 9.

39 Article 2 of the Data Protection Directive and Article 4 of the General Data Protection Regulation.

40 Article 29 of the Data Protection Working Party Group, Opinion 4/2007 on the concept of personal data, 01248/07/EN WP 136. In regards to rules applicable to collection, storage and use of tissue samples, the Working Party refers to Council of Europe Recommendation No. (2006)4 of the Committee of Ministers to Member States on research on biological materials of human origin.

41 Recital 34 in the preamble of the General Data Protection Regulation.

42 Svantesson, [26], p. 55

43 Article 4 of the Data Protection Directive containing the equivalent rule on territorial scope, is drafted somewhat differently since EU directives (but regulations) are to be implemented into national law. That article states: 1. Each Member State shall apply the national provisions it adopts pursuant to this Directive to the processing of personal data where: (a) the processing is carried out in the context of the activities of an establishment of the controller on the territory of the Member State; when the same controller is established on the territory of several Member States, he must take the necessary measures to ensure that each of these establishments complies with the obligations laid down by the national law applicable; (b) the controller is not established on the Member State”s territory, but in a place where its national law applies by virtue of international public law; (c) the controller is not established on Community territory and, for purposes of processing personal data makes use of equipment, automated or otherwise, situated on the territory of the said Member State, unless such equipment is used only for purposes of transit through the territory of the Community. 2. In the circumstances referred to in paragraph 1 (c), the controller must designate a representative established in the territory of that Member State, without prejudice to legal actions which could be initiated against the controller himself.

44 For definitions of the terms “controller” and “processor”, see Article 4 (7) and (8) in the General Data Protection Regulation.

45 See for a critical analysis, Svantesson, [26] p. 71.

46 Article 6.1(a) General Data Protection Regulation, corresponding to Article 7 Data Protection Directive. See also section 1 above.

47 Article 9 General Data Protection Regulation, corresponding to Article 8 Data Protection Directive. In regards to research, the regulation requires that appropriate safeguards are in place, which usually can consist of a review by a research ethics committee.

48 Corresponding to Article 25 in the Data Protection Directive.

49 Previously regulated in Article 26.2 in the Data Protection Directive, however the requirement to ensure that the rights of the data subject are enforceable was not explicitly mentioned. See further section 3.3.

50 Corresponding to Article 26.1 Data Protection Directive.

51 Safe Harbor Decision, [27].

52 C-362/14 *Schrems*, [5].

53 Ibid., para. 28.

54 Ibid., para. 73.

55 Ibid., paras. 85–86, referring to Safe Harbor Decision, Part B of Annex IV, [27].

56 Ibid., para. 94.

57 Ibid., para. 95.

58 MEMO 10/1661 of the European Commission, published on 3 December 2010, available at:
europa.eu/rapid/press-release_IP-10-1661_en.htm; MEMO 11/203 of the European Commission, published on 29 March 2011, available at: europa.eu/rapid/press-release_MEMO-11-203_en.htm and Communication from the Commission to the European Parliament and the Council Rebuilding Trust in EU U.S. Data Flows, COM(2013) 846 final and Communication from the Commission to the European Parliament and the Council on the Functioning of the Safe Harbour from the Perspective of EU Citizens and Companies established in the EU, COM(2013) 847 final.

59 See press release from the Commission; europa.eu/rapid/press-release_IP-16-216_en.htm?locale = en.

60 Communication from the Commission to the European Parliament and the Council, Transatlantic Data Flows: Restoring Trust through Strong Safeguards, COM (2016) 117 final.

61 Commission implementing decision of 12.7.2016 pursuant to Directive 95/46/EC of the European Parliament and of the Council on the adequacy of the protection provided by the EU-U.S. Privacy Shield, C (2016) 4176 final (EU/US Privacy Shield Decision).

62 Ibid., note 64, Annex II. The Principles are: Notice, Choice, Accountability for Onward Transfer, Security, Data Integrity and Purpose Limitation, Access and Recourse and Enforcement and Liability.

63 Ibid., recital 31 and Annex I and Annex II (Sec. I.3, Sec. I.4, III.6.d, and Sec. III.11.g)

64 Ibid.

65 Ibid., Recital 26 and supplemental principle “Dispute Resolution and Enforcement”, Annex II, Sec. III.11.

66 Ibid. Annex 2, Supplementary Principles, III.1.5.

67 Ibid. Annex 2, Overview, I.7.

68 Ibid., Annex 2, Supplementary Principles, III 1.5.c.

69 Ibid., Annex 2, Supplementary Principles, III 1.5.c. ii.

70 See for example, Mascalzoni, et al., [11] p. 721 and Int’l Soc’y for Biological & Envtl. Repositories, 2012 Best Practices for Repositories: Collection, Storage, Retrieval, and Distribution of Biological Materials for Research 147 (3d ed. 2011), *available at* isber.org/bp/documents/ISBERBestPractices3rdedition.pdf.

71 Mascalzoni, et al., [11] p. 724.

72 Article 26.2 Data Protection Directive and Article 46.3.a General Data Protection Regulation.

73 See for example, Mascalzoni, et al., [11] pp. 722, 724.

74 Article 46(1) General Data Protection Regulation.

75 According to Article 25(2) Data Protection Directive, the assessment is to be based on the following: The adequacy of the level of protection afforded by a third country shall be assessed in the light of all the circumstances surrounding a data transfer operation or set of data transfer operations; particular consideration shall be given to the nature of the data, the purpose and duration of the proposed processing operation or operations, the country of origin and country of final destination, the rules of law, both general and sectoral, in force in the third country in question and the professional rules and security measures which are complied with in that country.

76 C-362/14 *Schrems*, [5], para. 74.

77 In accordance with Article 52(1) in the EU Charter of Fundamental Rights and Hofmann, [28].

78 Ibid. p. 2 and C-362/14 *Schrems*, [5] paras. 94 and 95.

79 See Mascalzoni, et al., [11] pp. 726–727, where a draft MTA/DTA is provided.

80 Article 23.1 World Medical Association, Declaration of Helsinki - Ethical Principles for Medical Research Involving Human Subjects.

81
*Supra*, sections 3.2.2 and 3.2.3.

82 See above, section 3.2.3.

83 Dove, Knoppers, & Zawati, [4] p.6.

84 Information on the Global Alliance can beo found on its webpage, http://genomicsandhealth.org/.

85 See http://genomicsandhealth.org/about-the-global-alliance/mission-founding-principles, and Knoppers, [30].

86 Dove, [31] p. 679.

87 Svantesson, [26] where the author introduces the article by stating “Data privacy has emerged as a particularly fertile ground for transatlantic controversy.”

88 Kaye, [33] See also Thorogood & Zawati, [34], p. 699, with reference to Couzin-Frankel, [35] at pp. 502–503.

89 McHale, [13], p. 234 and Ruffert & Steinecke, [7] p. 98.

90 Article 50(c) in the General Data Protection Regulation authorized the Commission and the supervisory authorities in the Member States to engage in activities for international cooperation for the protection of personal data in relation to third countries, but unfortunately focuses merely on procedures for effective enforcement.
